# Assessing Hydrocyclone System’s Efficiency in Water-Borne Microplastics Capture Using Online Microscopy Sensors

**DOI:** 10.3390/s25030879

**Published:** 2025-01-31

**Authors:** Kacper Pajuro, Zhenyu Yang, Stefan Jespersen, Dennis Severin Hansen

**Affiliations:** Department of Energy, Aalborg University, Esbjerg Campus, Niels Bohrs Vej 8, 6700 Esbjerg, Denmark; kacperpajuro@gmail.com (K.P.); sje@energy.aau.dk (S.J.); dsh@energy.aau.dk (D.S.H.)

**Keywords:** microplastics, hydrocyclone, online microscopy, separation efficiency, statistic calibration, process control

## Abstract

Plastic pollution has been a global concern. Microplastics are often referred to as plastic particulates whose sizes are within the range of 1 μm to 5 mm. To cost-effectively capture these tiny microplastics from open environments, such as from the air or aquatic/marine systems, is far from trivial. Not only is some innovative capturing technology demanded, but some online monitoring solutions are often requested as well to assess the capturing effectiveness and efficiency, as well as provide some feedback information to the control system to adapt to varying operating conditions. Inspired by the de-oiling treatment of the produced water in offshore oil & gas production, this paper explores the potential to apply the hydrocyclone technology to cost-effectively handle the water-borne microplastics, and its effectiveness is demonstrated based on reliably calibrated online microscopy measurements subject to artificial polyethylene particulates added to the water stream. The experimental work is carried out using a commercial de-oiling hydrocyclone system and a set of commercial optical microscopy sensors. A statistic-based calibration method is firstly proposed for the deployed microscopy sensors to select the best calibration parameters. Afterwards these sensors are installed at the inlet and water-outlet of the hydrocyclone system via a side-stream sampling mechanism to assess this system’s (microplastics) separation efficiency subject to dynamical operating conditions, which are mimicked by manipulating its underflow and overflow control valves via PI-controlled loops. The separation efficiencies are calculated based on these volume concentration measurements and compared between the case with (statistically) optimal calibration parameters and the case with a set of non-optimal parameters. The best separation efficiency of 87.76% under the optimal calibration parameters is observed under a specific operating condition. The obtained result shows a promising potential to use these separation and sensing systems to cost-effectively handle aquatic microplastics collection, though it also indicates that a further higher efficiency could be achieved by some (microplastics) dedicated cyclone design combined with a dedicated process control system, and this is one part of our ongoing research work.

## 1. Introduction

A vast amount of plastic waste is being generated globally, along with the ever-growing demand for plastic products. It is estimated that about 6.3 gigatons (Gt) of plastic waste has been generated over the past 100 years. Among this waste, only 9% was recycled, 12% was incinerated, and 79% was collected in landfills or directly disposed of in the natural environment. According to the prediction in [1], by 2050, around 12 Gt of plastic waste could accumulate in the global environment.

Mismanaged plastic waste may end up in the oceans. Due to several factors, such as waves, currents, or radiant solar heat, plastic breaks down into smaller pieces in water. These plastic particles that are within the size range of 1 μm to 5 mm are often referred to as microplastics (MP) [2]. Due to their tiny sizes, microplastics can be unintentionally consumed by marine organisms such as zooplankton or fish [3]. Moreover, these particles can also be transferred to larger animals via the food chain [4]. Microplastics can also accumulate in humans, as their occurrence was observed in human feces [5]. It has been clear that lower-level organisms can be negatively affected by the accumulation of microplastics inside their bodies, as it may impact their growth or survival [6]. With respect to human beings, the contribution of microplastics to overall chemical intake is rather small [7], thereby the long-term impact of microplastics on our health is still disputable, and consequently, more research shall be conducted on this issue [8]. Nevertheless, being able to clean microplastics from the marine environment can be beneficial for long-term environmental protection as well as the reduction of health risks to human beings. There are two vital things that need to be considered to extract microplastics from water systems, i.e., some cost-effective capturing technologies as well as microplastics (online) measuring technologies [9].

Extensive research and lab-based instruments for measuring and analyzing microplastics have been developed in recent decades, such as the use of Raman spectroscopy, Fourier-transform infrared spectroscopy (FTIR), and light absorption technology [9]. However, little work can be found regarding in-field continuous (real-time) MP measurements. Depending on the kind of quantitative measurement index, besides the applied specific detection method/technology, the main challenge of online MP measurement also depends on the complicated features of MP and the carrying media, such as different MP sizes, shapes, and chemical compositions, as well as whether they are air-borne or water-borne, local point or wide open area, etc. Regarding the water-borne MP, the online fluorescence-based sensor could be applicable if the fluorescent dye can be added to the measurement stream, though some MP compositions possess a low affinity for fluorescent dye. The turbidity-based sensor could also be applied for concentration measurement if the aquatic turbidity variation is mainly the result of the dispersed MP compounds. The online (optical) microscopy-based sensor can also be applied for online MP concentration measurement if there is no desire to distinguish between different MP compositions (e.g., no mass concentration). The measured information and its accuracy depend on the selected sensing technology/instrument, application circumstances, and the user’s expectations. However, some reliable online measurements can not only provide dynamic information about the undergoing process, but also benefit the MP capturing process if the online MP measurement can be fed back to the process control system [10].

The currently common technology used to collect MP from aquatic systems is a type of filter-based filtration technique/system; however, the notorious fouling problem often needs to be carefully handled [11,12] in filter systems. Moreover, the filtration technology often comes with high costs and installation footprints. Inspired by previous work on the monitoring and control of de-oiling hydrocyclone process to clean the produced water in offshore oil & gas production [13,14,15,16,17], this work investigates the feasibility of applying a commercial (de-oiling) hydrocyclone system for MP capture from a point water resource. Following the physical separation principle, the hydrocyclone system can separate different liquid media from a continuous liquid phase based on their different densities using the centripetal force generated from a dedicated mechanical design and hydro-dynamical control [18]. Due to its high mechanical, thermal, and chemical robustness, small installation footprint, and extremely low CAPEX and OPEX (as well as the absence of chemical and bio materials), the hydrocyclone technology has been extensively used in various industrial applications.

To evaluate the MP capture efficiency of the considered hydrocyclone system, a set of commercial online microscopy sensors is deployed for online measurement of the MP volume concentration at the inlet and outlet of the hydrocyclone system. To guarantee reliable online MP measurements, a statistic-based calibration procedure is first proposed for the deployed microscopy sensor system. Furthermore, the hydrocyclone system is also controlled to mimic different flow conditions to find the optimal operating condition(s) that can lead to the highest capture efficiency. The experimental work conducted on a lab-scaled flow-loop system achieved the highest efficiency of 87.76% under dedicated hydrocylone operating conditions and the use of different sizes of artificial polyethylene particles.

The novel contributions of this paper lie in three folds: (i) a statistic-based calibration method is proposed, which combines different statistical features into the selection of the best calibration parameters for the deployed online microscopy systems; (ii) the separation efficiency of the deployed hydrocyclone system can be cost-effectively controlled by manipulating its underflow and overflow control valves, instead of making hardware retrofits, such as changing the hydrocyclone’s geometry and size, which can be economically expensive and time-consuming; finally, (iii) this study reveals that by properly controlling its operating flexibility, the deployed hydrocyclone system can still achieve a quite high MP capture efficiency, though it is initially designed to clean the water produced along with offshore oil and gas production [13,18].

Some relevant literature reported that the MP removal efficiency can be lifted to over 99% for some dedicated mini-hydrocyclones and specific MP compositions [19,20,21]. This also indicates that the efficiency of the hydrocyclone system used in this study could be further improved and kept robust by proper geometric and mechanical retrofits, along with a good process control solution. The differences between this study and the aforementioned literature lie in two perspectives: (i) the separation efficiency was assessed by various offline methods in those studies while the online sensor is used in this study. On one hand, the offline approaches could lead to more accurate measurement at the steady operation, but on the other hand, these methods cannot be used for assessing dynamic behaviors due to lack of real-time capability. (ii) The operational efficiency of a hydrocyclone system not only depends on its feeding flow rate but is also heavily impacted by its pressure-drop-ratio (PDR) [13,16,17,18], which is defined as the ratio of the differential pressure between the overflow and inlet over the differential pressure between the underflow and inlet. Thereby, both the PDR control and flow control are coordinated in this study by controlling the overflow and underflow control valves (in addition to controlling the supply pump speed), while the other literature focused only on the flow control to emulate different operating conditions.

## 2. Materials and Methods

### 2.1. MP Particles for Calibration and Testing

Two sets of polystyrene micro-beads manufactured by BS-Partikel are used as calibration particles, and they are supposed to follow the normal distribution as stated by the manufacturer:

- mean diameter $\mu_{BS}$—40.3 μm, and the Standard Deviation (STD) $\sigma_{BS}$—0.89 μm;
- mean diameter $\mu_{valBS}$—79.4 μm, and the STD $\sigma_{valBS}$—1.75 μm.

**Figure 1:**
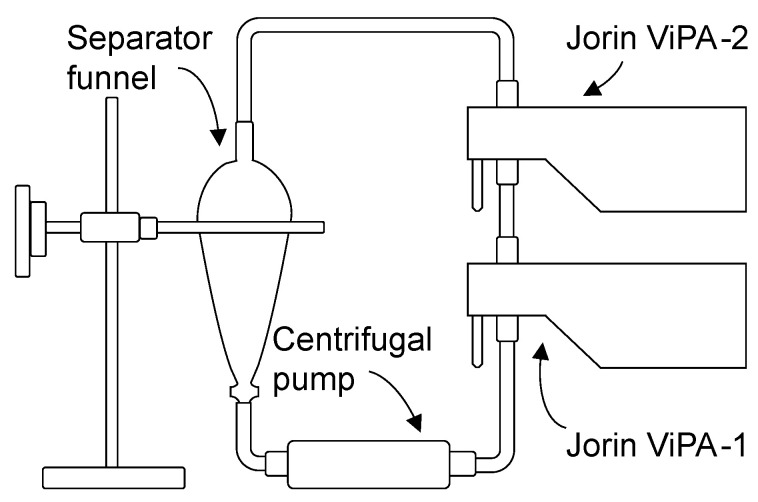
Diagram of ViPA calibration flow-loop.

A total of 50 g of calibration particles are mixed with water to prepare the calibration fluid that circulates through the calibration setup, as shown in Figure 1. Approximately 80% of added micro-beads are small, and the remaining 20% are large.

For the cyclone-based separation experiments, the red polyethylene (PE) micro-spheres produced by Cospheric LLC, with a density of 0.98 g/cc and two different size ranges of 53–63 μm and 70–90 μm, are used. Approximately 30 g of small micro-spheres and 10 g of large ones are mixed with 170 L of tap water in the mixture tank.

### 2.2. Microscopy Sensing System

The online microscopy sensing system used in this study is a commercial product named Visual Process Analyser (ViPA), manufactured by Process Imaging Limited (former Jorin Limited). The sensing unit contains a high-resolution digital video camera on one side of a flow cell and a light source on the other side. The built-in software performs a dedicated image processing algorithm based on obtained sequential data to provide quantitative information, such as the number of detected particles, their sizes or volumes, and thereby the (volumetric) concentration measurement. The highest frequency of measurement update is one Hertz. The key technical parameters of ViPA sensors are presented in Table 1 [14].

### 2.3. Calibration Setup and Statistic-Based Calibration Method

A dedicated calibration method based on diverse statistical tests is proposed in this study, and the calibration data is generated based on the calibration setup illustrated in Figure 1. Two ViPAs were connected in series, and a mixture of demineralized water and calibration (BS) particles circulated through this flow loop via the centrifugal pump with a constant flow rate.

There are two (user-oriented) tuning parameters when calibrating the ViPA system:

- The edge-strength-value (ESV) of a detected particle is defined as the gradient in the grey scale from the background at its edges. The higher the ESV, the sharper the detected object appears to be in a frame at the number range of 0–10. The manufacturer of the ViPA sensor does not specify the method behind calculating the ESV; however, it is presumed that some version of the Sobel filter is applied.
- The threshold-value (TV) determines the level of darkness required for the pixels of a particle to be detected at the number range of 0–255. The higher the TV, the darker the particle needs to be in order to be classified as one.

**Figure 2:**
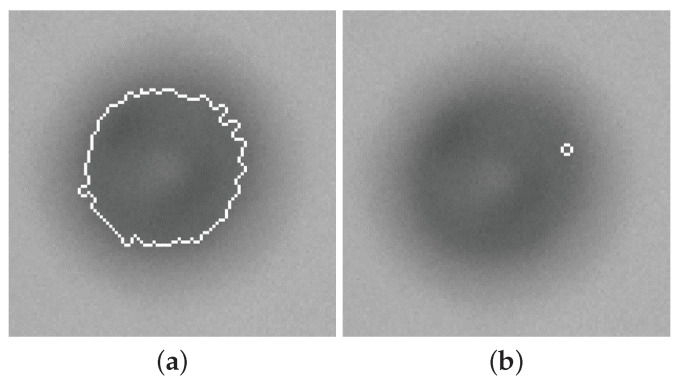
Liberal (**a**) ESV = 1 vs. Strict (**b**) SV = 2. Both with the same TV = 62 on the same frame [14].

The proper choices of these parameters can significantly affect the detection and measurement of existing particles and their overall quantities. For example, two different cases, named Liberal and Strict scenarios, are illustrated in Figure 2, where the selected edge is visualized with a white contour.

Normally, the ESV and TV are recommended based on visual inspection of a number of images of calibration particles. Even if the white contour may fit relatively well to manually analyzed particles, calibration settings may be inaccurate overall, as the degree of haziness varies substantially throughout all captured images. Therefore, a more reliable calibration method is required. In this paper, we propose a statistic-based calibration method through which the optimal ESV and TV values are chosen with respect to the minimal variation of selected statistical features based on the extensive calibration experimental data rather than human perception. We would like to note that the application of cutting-edge image processing algorithms is beyond the scope of this study.

The original experimental (image) data obtained by ViPA systems is processed according to different possible combinations of ESV and TV choices by applying the ViPA built-in software. The ESV is chosen from {0,1,2,…,10}, and TV is chosen from {30,31,32,…,90}, which in total adds up to 620 different combinations. The obtained data from different cases are assessed based on their proximity to the dedicated distribution feature claimed by calibration BS particles through committing dedicated statistic tests, such as the z-test, Kolmogorov–Smirnov test, chi-square test, Kullback–Leibler divergence, Jensen–Shannon divergence, and sum of squared errors. The best combinations w.r.t. different testing criteria are further validated using the measured data regarding the large particles ($\mu_{valBS}$).

### 2.4. Hydrocyclone System and Its Control

During this study, an off-the-shelf commercial hydrocyclone liner (Vortoil D35) produced by Schlumberger is used. Vortoil D35 was initially designed as a deoiling hydrocyclone for produced water treatment in oil and gas production. More information about this type of system and its de-oiling application can be found in [13,15,16,17]. The Hydrocyclone performs separation based on density differences of a mixture. It consists of an inlet, through which a mixture is injected into the cylinder segment. A type of vortex can be created inside the hydrocyclone chamber due to the centripetal force; thereby, the lighter phase moves towards the center of the chamber to form an air/oil rich core, then this core stream is repelled out of the hydrocyclone via its overflow outlet. The heavier phase moves closer to the wall and exits the hydrocyclone via its underflow outlet. Different from the typical membrane filtration process where the rejected compound(s) often accumulate upon the filter’s surface, which naturally causes the fouling problem [11], the hydrocyclone system collects the rejected compound at its overflow outlet, while the cleaned stream flows through the hydrocyclone without any fouling-induced resistance [13,18].

**Figure 3:**
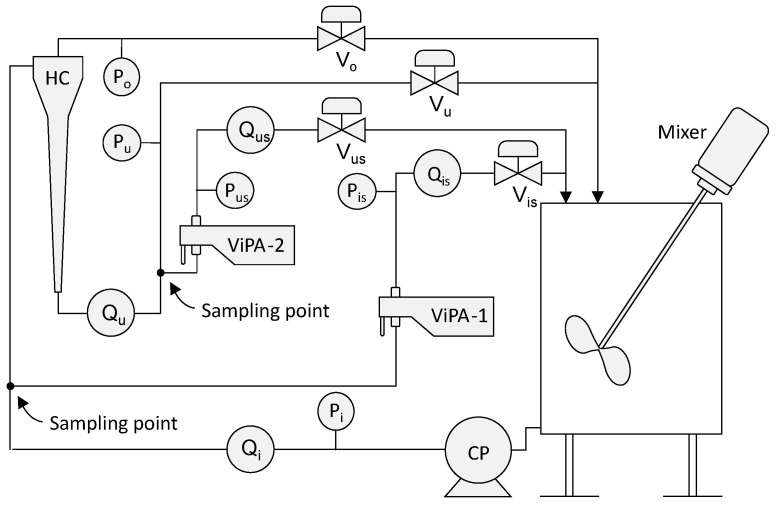
Diagram of hydrocyclone flow-loop setup.

A testing flow loop is constructed at our laboratory. The schematic diagram of the setup is shown in Figure 3. Two sampling side streams are adopted in this configuration to guarantee the flow rates over these two installed ViPA sensors within the specified condition. Sensor ViPA-1 is used to measure the inlet’s particle concentration, whereas sensor ViPA-2 measures the underflow’s particle concentration. Consequently, the MP capture efficiency can be calculated accordingly. The subscript *i* indicates the hydrocyclone’s inlet relevance, *u* indicates the hydrocyclone’s underflow relevance, *o* indicates the hydrocyclone’s overflow relevance, and us indicates the underflow’s side-stream relevance, is indicates the inlet’s side-stream relevance. A mechanic mixer is installed in the circulation tank to prevent any undesired separation of micro-beads from water inside the tank due to the gravity impact.

Besides these ViPA sensors, a set of pressure sensors denoted as *P* and flow meters denoted as *Q* is also installed in this setup to monitor and control the hydrocyclone’s operating condition. For example, these pressure measurements $P_{i}$, $P_{u}$, and $P_{o}$ are utilized to calculate the PDR according to Equation (1), which is one of the key operating parameters for hydrocyclone operation [13,18]. Moreover, it is often used to control the hydrocyclone system to maintain its adequate separation efficiency, which is denoted as *e* here and is defined as Equation (2), where $C_{u}$ and $C_{i}$ represent the volume concentration at the underflow and inlet, respectively.

(1) $$PDR=\frac{P_{i}-P_{o}}{P_{i}-P_{u}}.$$

(2) $$e=1-\frac{C_{u}}{C_{i}}.$$

A number of control valves, denoted as *V*, are installed to manipulate different operating conditions. By varying the opening degrees of the underflow $V_{u}$ and overflow $V_{o}$ valves, a set of experiments is conducted. During the experiment, the opening degree of $V_{u}$ increased from 50% to 100% step-by-step with a step size of 5%. During each step of $V_{u}$’s opening degree, the opening degree of $V_{o}$ changes from 0% to 50% with a piece-wise step size of 5%. This procedure results in 121 piece-wise constant segments of the opening degrees of $V_{o}$ and $V_{u}$. The duration of each segment is about 5 min. One whole experiment took about 10 h and 10 min. The opening degrees of valves $V_{us}$ and $V_{is}$ are individually controlled by a developed PI controller using flow-rate feedback from $Q_{us}$ and $Q_{is}$, respectively, to maintain a proper side-stream flow rate through individual ViPA sensors.

## 3. Results

### 3.1. Results of Calibration Experiment

First of all, all outliers with 5 $\sigma_{BS}$ away from $\mu_{BS}$ from all scenarios are removed, and the remaining data is also filtered via a low-pass filter. Then, the z-test is applied to find the qualified pair of ESV and TV values that lead to the “true” proximity to the mean size of $\mu_{BS}$ stated in the data-sheet. For all scenarios, two-sided tests with 95% confidence level were conducted. Test statistics *z* were calculated based on the individual sample mean $\hat{\mu }$, according to Equation (3):

(3) $$z=\frac{\hat{\mu }-\mu_{BS}}{\sigma_{BS}/\sqrt{n}}$$

**Figure 4:**
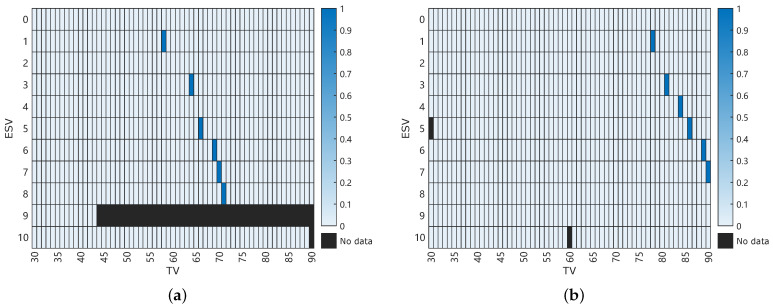
Calibration settings (blue) with |z|<1.96 and n>1000 for ViPA-1 (**a**) and ViPA-2 (**b**).

Concerning the impact of the number of detected particles *n*, an additional requirement of n>1000 was imposed. All scenarios that satisfy both aforementioned requirements are marked in blue in Figure 4.

It can be observed that six different sets of calibration settings met the aforementioned requirements for each sensor setup; thus, they are considered potential candidates for the best calibration settings.

Besides the z-test of the proximity of $\hat{\mu }$ to $\mu_{BS}$, all potential (best) candidates are further analyzed using other statistical criteria in the following.

#### 3.1.1. Kolmogorov–Smirnov Test

Based on the measurement data from the scenarios that pass the z-test, the statistical cumulative distribution functions (CDF) can be constructed and compared with the “true” CDF based on the data-sheet of $\mu_{BS}$ and $\sigma_{BS}$ through the Kolmogorov–Smirnov test (KS-test). This statistical test investigates whether the statistical distribution $\hat{CDF}\left(x\right)$ is consistent with the hypothesized $CDF_{BS}\left(x\right)$ by evaluating the statistic distance $d_{KS}$ as [22]:

(4) $$d_{KS}=\max_{x}\left(\right|\hat{CDF}\left(x\right)-CDF_{BS}\left(x\right)\left|\right)$$

For these CDFs to be considered consistent, the $d_{KS}$ needs to be smaller than $1.36/\sqrt{n}$. As n>1000 is a pre-condition for all potential best candidates, none of the remaining scenarios can pass the KS-test. Nevertheless, we still adopt the index $d_{KS}$ as a part of comparable measure of goodness-of-fit; therefore, the $d_{KS}$ values for six best scenarios for both sensors are listed in Table 2.

#### 3.1.2. Chi-Square Test

The consistency of the obtained sample standard deviation *s* compared with the “true” $\sigma_{BS}$ from the data-sheet is also committed via the chi-square test. In order to mitigate the impact of the large number of *n*, the denormalized and decentralized $T_{norm}$ is adopted as described in Equation (5). The top six scenarios with the smallest $T_{norm}$ for both sensors are listed in Table 3.

(5) $$T_{norm}=|1-\frac{T}{n-1}|\;=|1-\frac{s^{2}}{\sigma_{BS}^{2}}|$$

#### 3.1.3. Kullback–Leibler Divergence

The statistical test, named Kullback–Leibler (KL) divergence, denoted as $D_{KL}\left(P||Q\right)$ in Equation (6), is also applied to quantify the statistical distance between data-based P(x) and model Q(x) in terms of probability distributions [23]:

(6) $$D_{KL}\left(P||Q\right)=\sum_{x}P\left(x\right)\log\left(\frac{P(x)}{Q(x)}\right).$$

To apply this calculation, the measured calibration data were grouped into bins with the bin width equaling the length of one pixel, i.e., 0.375 μm. Based on this statistical distance, the best candidates of ESV and TV pairs are presented in Table 4. For some scenarios, $D_{KL}\left(P||Q\right)$ cannot be computed due to some empty bin in sampled P(x).

#### 3.1.4. Jensen–Shannon Divergence

Because KL divergence is not a symmetrical measure of statistical difference, as $D_{KL}\left(P||Q\right)\neq D_{KL}\left(Q||P\right)$, the best order listed in Table 4 would be different if they were sorted according to $D_{KL}\left(Q||P\right)$. Therefore, the symmetrical version of KS divergence, known as Jensen–Shannon (JS) divergence, is also calculated according to Equations (7) and (8) [24]:

(7) $$D_{JS}\left(P||Q\right)=\frac{1}{2}D_{KL}\left(P||M\right)+\frac{1}{2}D_{KL}\left(Q||M\right)$$

(8) $$M=\frac{1}{2}\left(P+Q\right)$$

Based on $D_{JS}\left(P||Q\right)$, the best scenarios are listed in Table 5. Similarly as for KL divergence, some JS divergence could not be calculated due to empty bin(s) in sampled P(x).

#### 3.1.5. Sum of Squared Errors

To mitigate the requirement of absolute continuity of probability distributions for KL and JS divergences, an alternative approach using sum of squared errors (SSE) between P(x) and Q(x) is calculated for all scenarios as:

(9) $$SSE=\sum_{x}{\left(P\left(x\right)-Q\left(x\right)\right)}^{2}.$$

The best 6 scenarios with least SSEs are listed in Table 6.

#### 3.1.6. Assessment of Different Statistical Criteria

Best ESV and TV pairs are selected according to different statistical criteria as listed in Table 7.

It is quite clear that for ViPA-2 sensor, the best ESV and TV pair suggested by different criteria lies in ESV of 4 and TV of 84. Therefore, this calibration set is chosen in the following for ViPA-2. There is no unanimity in results for the ViPA-1 sensor. While SEE criterion suggests ESV of 1 and TV of 58, all other 4 out of 5 criteria propose ESV of 3 and TV of 64. Therefore, the skewness of different criteria are also calculated by Equation (10), where $x_{i}$ is the size of the *i*th detected particle in an experiment. The skewness of a perfectly symmetrical distribution equals 0, and as asymmetry increases, so does the absolute value of skewness [25].

(10) $$skewness=\frac{\sqrt{n(n-1)}}{n-2}\frac{\frac{1}{n}\sum_{i=1}^{n}{\left(x_{i}-\hat{\mu }\right)}^{3}}{{\sqrt{\frac{1}{n}\sum_{i=1}^{n}{\left(x_{i}-\hat{\mu }\right)}^{2}}}^{3}}.$$

According to Table 7, the scenario corresponding to ESV of 3 and TV of 64 leads to a more symmetrical distribution. Therefore, this setting is chosen as the final calibration set for the ViPA-1 sensor.

**Figure 5:**
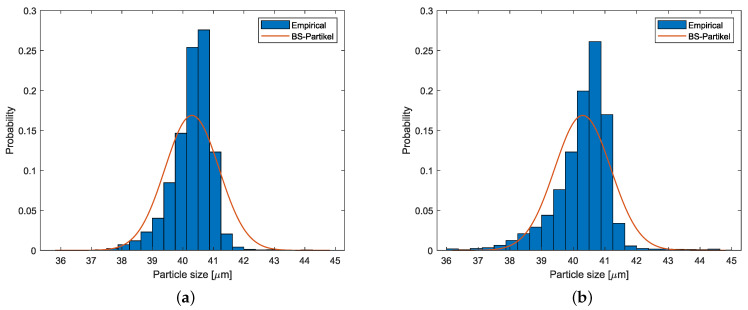
Sampled PDF for ViPA-1 with ESV: 3, TV: 64 (**a**), and for ViPA-2 with ESV: 4, TV: 84 (**b**).

The probability distributions P(x) for the best final calibration settings for both sensors are presented in Figure 5. These histograms are compared to the “true” normal distribution provided by the vendor with parameters $\mu_{BS}$ and $\sigma_{BS}$, which is marked in red in Figure 5.

#### 3.1.7. Validation of Calibration Settings

**Figure 6:**
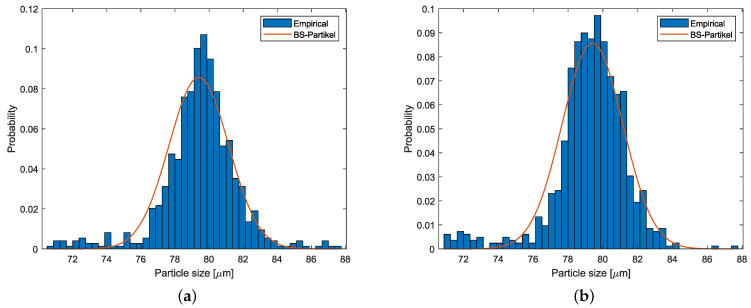
Validation probabilities for ViPA-1, ESV: 3, TV: 64 (**a**), and for ViPA-2, ESV: 4, TV: 84 (**b**).

The best calibration settings are further checked to ensure their versatility in estimating different particle sizes. This validation procedure is committed based on large polystyrene particles with $\mu_{valBS}$ and $\sigma_{valBS}$. By applying the best calibration settings for both sensors, analysis of large size measurement was performed. The probability histograms of large particles, within the size range of 5 $\sigma_{valBS}$ away from $\mu_{valBS}$, are presented in Figure 6. The the width of the bin 0.375 μm remains unchanged. The red plot represents the true normal distribution of the validation particles.

Concerning the large size of calibration particles, the probability bins of detected particles follow the desired pattern well. Along with the visual verification, numerical validation was performed by computing the z-test for both sensors. Based on the corresponding $\hat{\mu }$ and *n*, the following *z* statistics were calculated: ViPA-1: z=1.09 and ViPA-2: z=2. For the 99% confidence level, both *z* values were smaller than the absolute critical value of 2.58. Thus, the null hypothesis was accepted for both sensors, which further validates the appropriate performance of the best calibration settings.

**Figure 7:**
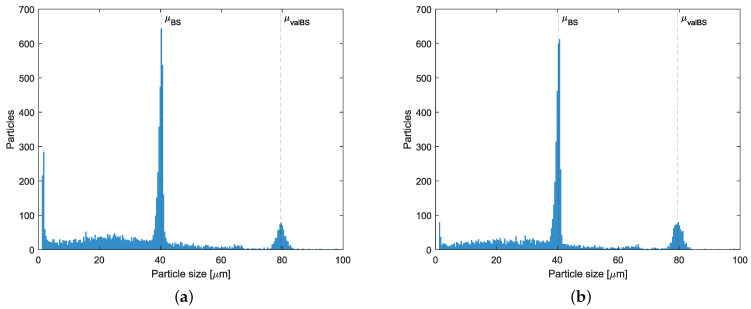
Histogram of all detected particles for ViPA-1, ESV: 3, TV: 64 (**a**), and for ViPA-2, ESV: 4, TV: 84 (**b**).

The quantity of particles detected outside the aforementioned calibration and validation size ranges was captured and analyzed. Size histograms of all detected particles are presented in Figure 7. As expected, a relatively large number of particles were observed in proximity to $\mu_{BS}$ or $\mu_{valBS}$. However, particles significantly smaller than $\mu_{BS}$ are also observed by both sensors; this could be explained by some already existing debris (like miniature particles) in the tap water. To distinguish these particulates, more parameters in the image analysis are needed, which is beyond the scope of this paper but is a topic for our future work.

### 3.2. Results of Hydrocyclone MP-Separation Experiment

**Figure 8:**
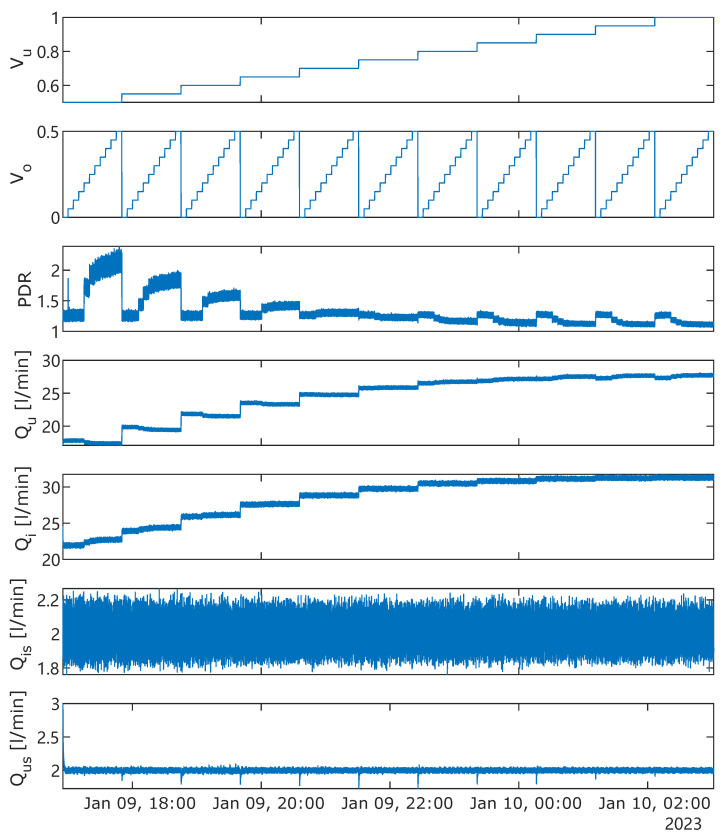
Measurement from one complete testing using hydrocyclone setup.

A set of testing experiments using the de-oiling hydrocyclone setup is designed as described in Section 2.4. The best calibration settings of both ViPA sensors are adopted. There are two manipulated variables (MVs) corresponding to the opening degrees of both control valves $V_{u}$ and $V_{o}$, respectively. The controlled variables (CVs) are the PDR and (MP-) separation efficiency. The flow rates at the inlet $Q_{i}$, outlet $Q_{u}$, and both sampling stream $Q_{is}$ and $Q_{us}$, are also measured. The data for one complete test lasted for 10 h and 10 min are presented in Figure 8.

**Figure 9:**
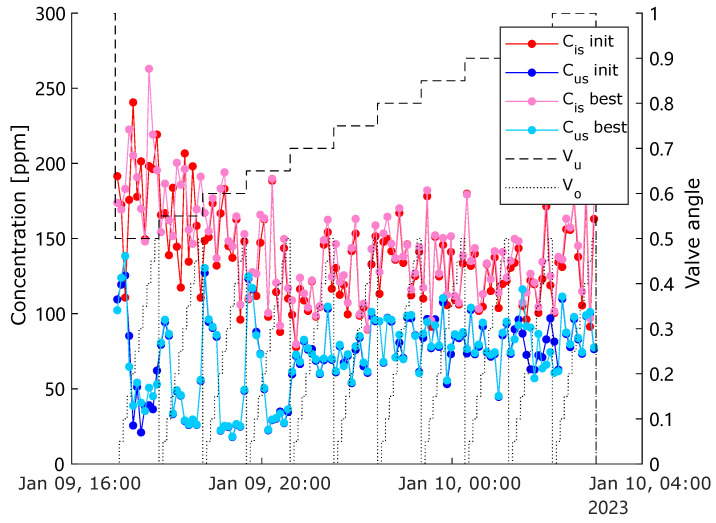
Mean concentration measurements for one complete experiment using the best calibration setting compared with one non-optimal setting “init”.

To avoid the transient dynamics caused by switching the opening degrees of the control valves, the measured data within the first 5% and last 5% of each segment duration are disregarded, and all sensors, except ViPA sensors, were performing measurements simultaneously, with the same sampling rate (100 Hz). All primary data measured by both ViPA sensors are first saved in tabular format, and then the characteristic properties describing the detected particles are derived through the sensor’s built-in image processing software. The volumetric concentration of each frame is thereby computed. For simple illustration, the mean concentration for each combination of different opening degrees of Vu and Vo is presented in Figure 9, where $C_{is}$ is the mean value of ViPA-1 measured concentration, and $C_{us}$ is the mean of ViPA-2’s measurement.

Although $Q_{is}$ was maintained at the same level by its local control loop, as shown in Figure 8, it can still be observed that the concentration $C_{is}$ of the inlet sample stream gradually decreased over time. We suspect that this could be caused by micro-beads adhering to the surfaces and corners of the tank and flow pipelines as the experiment goes. Nevertheless, it is still believed that the measured concentrations $C_{is}$ and $C_{us}$ via sampling loops are considered reasonable approximations of the corresponding mainstream concentrations of $C_{u}$ and $C_{i}$, respectively. This means that we assumed that the T-junction did not significantly impact the proportions of MP concentrations in the main stream and the side stream, though this assumption might need to be validated [14].

**Figure 10:**
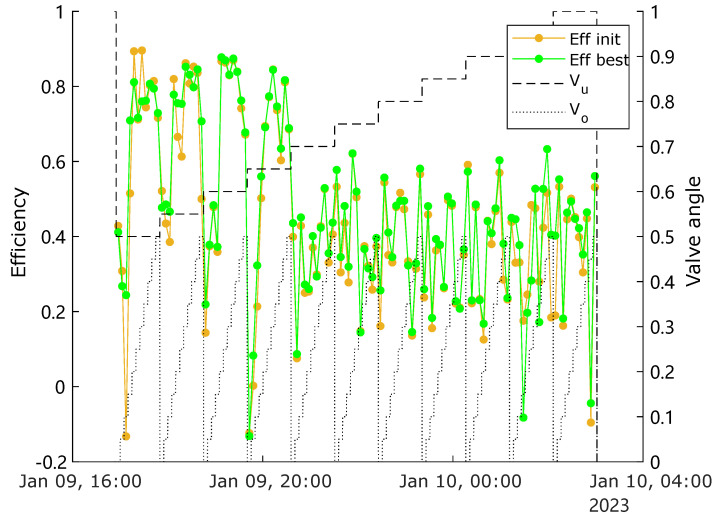
Mean-efficiencies for different (MV) operating conditions compared between the best and the non-optimal calibration settings.

The mean efficiency throughout the entire experiment is presented in Figure 10, where the result from the best calibration setting is also compared with the result from a non-optimal calibration setting. It can be observed that the best calibration resulted in smaller oscillation amplitudes compared with the scenario using the non-optimal setting, which indicates a better reliability and robustness in usage of the optimal parameters.

**Figure 11:**
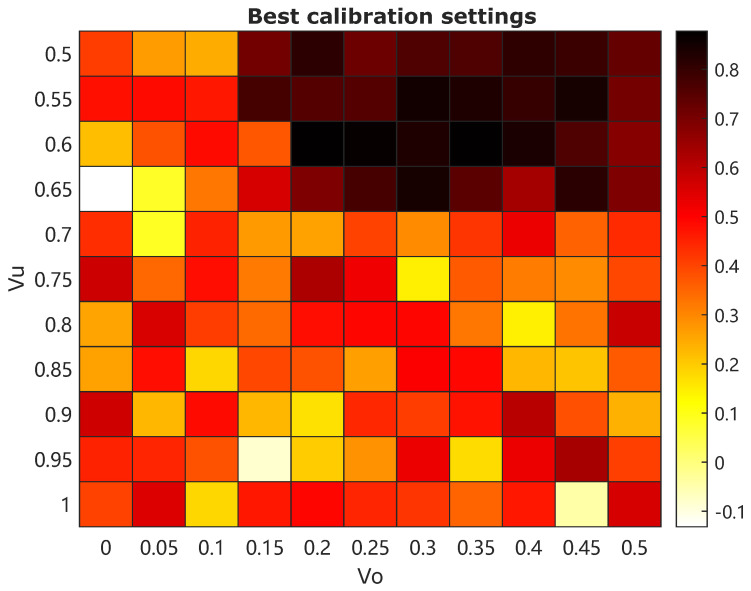
Separation efficiency heat-map subject to the best calibration settings.

The efficiencies corresponding to each pair of $V_{u}$ and $V_{o}$ segments are depicted in the heat-map in Figure 11. It can be observed that a high separation efficiency, e.g., above 80%, can be achieved in the following region of operation for valve opening degrees: $V_{u}:\;0.5-0.65$ and $V_{o}:\;0.2-0.45$. It can be noted that a value of $V_{u}$ higher than 0.45 leads to decreased efficiency, as the inlet flow $Q_{i}$ may be too fast, which results in the disruption of the vortex core generation of the lighter phase (MP-rich stream). Furthermore, decreased efficiency is also observed for a value of $V_{o}$ smaller than 0.2, as the overflow channel may be too narrow to sufficiently allow the separated MP-rich stream to exit through the overflow channel. Therefore, some proper control of the hydrocyclone system is crucial in maintaining a high separation efficiency subject to diverse operating conditions and disturbances.

#### 3.2.1. Separation Efficiency vs. Control Parameters

**Figure 12:**
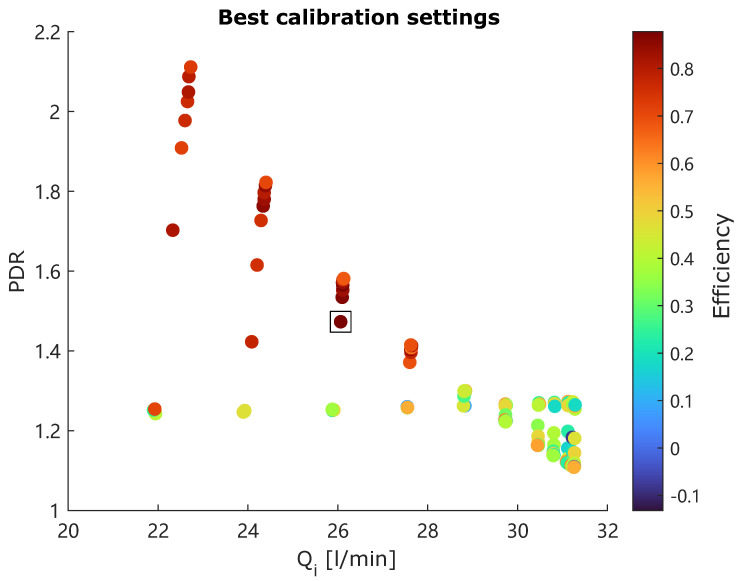
Efficiency vs. $Q_{i}$ and PDR subject to the best calibration setting.

The robustness and stability of a hydrocyclone-based operation are usually ensured through proper feedback control of PDR and inlet flow $Q_{i}$ [13,15,17,18]. Therefore, the obtained efficiencies are also compared with the corresponding PDR and $Q_{i}$, as illustrated in Figure 12.

It is clear that the controllable variables should be maintained at the following levels:

- PDR: 1.4–2.2, and
- $Q_{i}$: 22–28 L/min,

It is possible to maintain a high separation efficiency using the current hydrocyclone setup, though it was initially designed for de-oiling purposes applied in offshore produced water treatment [13,15,16,17]. The highest observed efficiency of 87.76% is depicted by a square in Figure 12. It can be achieved with the PDR value of 1.5 and $Q_{i}$ of 26 L/min. it should be noticed that these observed preferable values are in the line with recommendations from the manufacturer of this hydrocyclone system.

#### 3.2.2. Size Impact to Separation Performance

**Figure 13:**
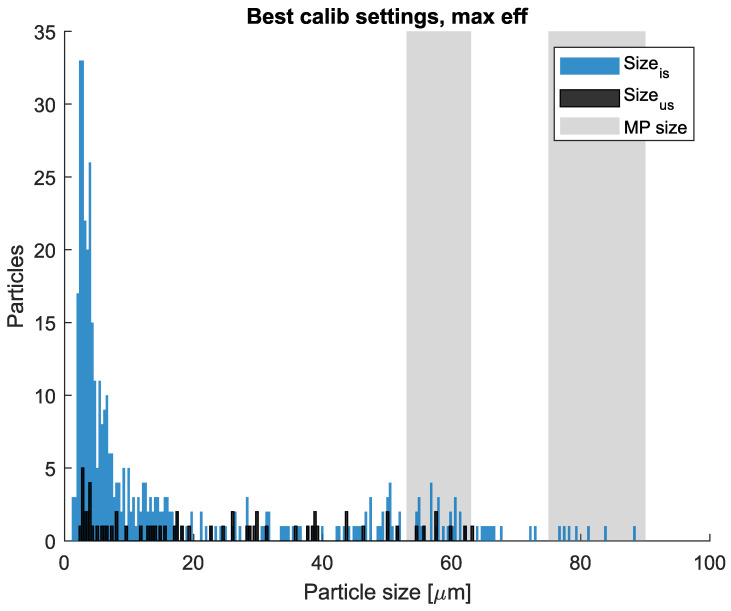
Histogram of detected particles for the best efficiency segment subject to the best calibration setting.

It is well known that the particle size can play a vital rule in determining a hydrocyclone’s separation efficiency [13,14,18]. Therefore, the hydrocyclone performance against different particle sizes is also explored based on the measured data. By focusing on the most efficient segment of operation, the size histogram of all detected particles is presented in Figure 13. The gray areas in the plot correspond to the aforementioned size ranges provided by the manufacturer of micro-spheres. The blue color indicates the number of particles observed in the inlet sampling stream, whereas the black color is associated with the observed particles in the underflow sampling stream.

For the large-sized micro-spheres, no particles were spotted in the underflow sampling stream. This indicates 100% efficiency of separation for these large microplastics, though they only account for a small portion of total micro-spheres. Although small-sized micro-spheres are still detected in the underflow sampling stream, the vast majority of these particles are separated when compared with the inlet measurement. This observation is also in line with the separation principle of a hydrocyclone system, i.e., it is easier to separate larger particles subject to the same density and surface characteristics.

It can be clearly observed that the majority of particles detected by both sensors have significantly smaller sizes than the ranges of added micro-spheres, though their contributions to the overall volumetric concentration can be negligible. This could be due to a number of reasons, such as (i) the tap water is not clean enough; (ii) some small debris broken from the tank and flow pipelines, as well as from the control valves or circulation pump etc., due to corrosion or erosion inside the testing setup; (iii) micro-spheres could be sheared into smaller pieces due to the standard centrifugal circulation pump, T-shaped sampling mechanism, and/or the shearing impact from the mixture propellers; (iv) the self degradation of micro-spheres due to the external shearing and temperature impacts; or (v) simply fake measurements due to the sensor’s hardware and software limitation. Investigating these possible reasons for this observed phenomenon will be part of our future work.

## 4. Conclusions

This work investigated the effectiveness and efficiency of capturing microplastics from the aquatic system using the hydrocyclone system and online microscopy sensors. To be sure that the online microscopy sensors provide reliable and accurate measurements, a statistical calibration method is proposed based on different statistical indices, instead of just following a non-statistical and subjective approach. The calibration parameters of ESV and TV provided, along with the selected commercial ViPA sensors, are carefully selected after sufficient statistical analysis based on total 620 data scenarios for both ViPA sensors. The best calibration setting is also validated using other calibration particles of different size. This systematical approach can help make applications of online microscopy sensors more reliable and confident with the data obtained. We also believe that the proposed statistic-based calibration method could also be applicable for general online microscopy calibration as well.

The effectiveness and efficiency of a type of de-oiling hydrocyclone system for capturing microplastics from water systems is further assessed using the calibrated online microscopy sensors. It can be clearly observed from Figure 10 and Figure 11 that the applied hydrocyclone system can effectively remove microplastics from the water system, subject to proper operating conditions, and the separation efficiency can be achieved up to 87.76% during our study, though this hydrocyclone system is originally designed for de-oiling for offshore produced water treatment. Of course, this type of de-oiling hydrocyclone system is not suitable for capturing microplastics with densities heavier than water or in a scenario where the microplastics concentration at the feeding flow is too high (e.g., over 1%). Some other types of hydrocyclone/cyclone systems, such as de-sanding cyclone systems or dedicated hydrocyclones, need to be investigated for effective capture of “heavy” microplastics [10,20,21]. The combination of the design of some dedicated hydrocyclone/cyclone system, together with smart process control, e.g., AI-based control solutions [17], could be an interesting topic in our future work.

## Figures and Tables

**Table 1:** ViPA’s key specifications.

	Jorin ViPA
Particle size range	1.2–150 μm
Concentration	0–2500 ppmV
Inlet and outlet ports	1/4″
Flow rate	up to 4 L/min
Max operating pressure	120 bar
Frame rate	∼30 fps
Pixel length conversion factor	0.375 μm/pixel
Pixel area conversion factor	0.1406 μm^2^/pixel

**Table 2:** Best ESV and TV combinations according to index $d_{KS}$ for ViPA-1 (**a**) and ViPA-2 (**b**).

(a)				(b)			
**Rank**	**ESV**	**TV**	$d_{\mathrm{KS}}$	**Rank**	**ESV**	**TV**	$d_{\mathrm{KS}}$
1	3	64	0.1302	1	4	84	0.1125
2	1	58	0.1460	2	5	86	0.1171
3	5	66	0.1639	3	3	81	0.1449
4	6	69	0.1854	4	6	89	0.1525
5	7	70	0.2256	5	7	90	0.1776
6	8	71	0.2357	6	1	78	0.1890

**Table 3:** Best performing experiments according to $T_{norm}$ for ViPA-1 (**a**) and ViPA-2 (**b**).

(a)				(b)			
**Rank**	**ESV**	**TV**	$T_{\mathrm{norm}}$	**Rank**	**ESV**	**TV**	$T_{\mathrm{norm}}$
1	3	64	0.4040	1	4	84	0.0544
2	5	66	0.5542	2	5	86	0.3063
3	6	69	0.6622	3	6	89	0.4020
4	7	70	0.7744	4	3	81	0.4063
5	8	71	0.7871	5	7	90	0.5091
6	1	58	0.9770	6	1	78	1.0298

**Table 4:** Best ESV and TV pairs according to $D_{KL}\left(P||Q\right)$ for ViPA-1 (**a**) and ViPA-2 (**b**).

(a)				(b)			
**Rank**	**ESV**	**TV**	$D_{\mathrm{KL}}\left(P||Q\right)$	**Rank**	**ESV**	**TV**	$D_{\mathrm{KL}}\left(P||Q\right)$
1	3	64	0.1582	1	4	84	0.1551
2	5	66	0.2618	2	5	86	0.1843
3	1	58	0.3194	3	3	81	0.2125
4	6	69	-	4	6	89	0.2231
5	7	70	-	5	1	78	0.3856
6	8	71	-	6	7	90	-

**Table 5:** Best ESV and TV pairs according to $D_{JS}\left(P||Q\right)$ for ViPA-1 (**a**) and ViPA-2 (**b**).

(a)				(b)			
**Rank**	**ESV**	**TV**	$D_{\mathrm{JS}}\left(P||Q\right)$	**Rank**	**ESV**	**TV**	$D_{\mathrm{JS}}\left(P||Q\right)$
1	3	64	0.0420	1	4	84	0.0356
2	1	58	0.0439	2	3	81	0.0419
3	5	66	0.0706	3	5	86	0.0443
4	6	69	-	4	6	89	0.0568
5	7	70	-	5	1	78	0.0574
6	8	71	-	6	7	90	-

**Table 6:** Best ESV and TV pairs according to SSE for ViPA-1 (**a**) and ViPA-2 (**b**).

(a)				(b)			
**Rank**	**ESV**	**TV**	SSE	**Rank**	**ESV**	**TV**	SSE
1	1	58	0.0208	1	4	84	0.0226
2	3	64	0.0297	2	3	81	0.0246
3	5	66	0.0478	3	5	86	0.0293
4	6	69	0.0687	4	6	89	0.0325
5	7	70	0.1037	5	1	78	0.0327
6	8	71	0.1210	6	7	90	0.0528

**Table 7:** Best ESV and TV pairs with respect to different criteria for ViPA-1 (**a**) and ViPA-2 (**b**).

(a)				(b)			
**Criterion**	**ESV**	**TV**	**Skewness**	**Criterion**	**ESV**	**TV**	**Skewness**
KS-test	3	64	−0.7046	KS-test	4	84	−0.7356
Chi-test	3	64	−0.7046	Chi-test	4	84	−0.7356
KL divergence	3	64	−0.7046	KL divergence	4	84	−0.7356
JS divergence	3	64	−0.7046	JS divergence	4	84	−0.7356
SSE	1	58	−1.1373	SSE	4	84	−0.7356

## Data Availability

Data available on request from the authors.

## Abbreviations

The following abbreviations are used in this manuscript:

ViPA	Visual Process Analyser
$\mu_{BS}$	Mean particle size provided by manufacturer BS-Partikel
$\mu_{valBS}$	Mean validation particle size provided by manufacturer BS-Partikel
$\sigma_{BS}$	Standard deviation of particles provided by manufacturer BS-Partikel
$\sigma_{valBS}$	Standard deviation of validation particles provided by manufacturer BS-Partikel
DoF	Depth of Field
ESV	Edge Strength Value
TV	Threshold Value
_*i*_	Inlet part
_*u*_	Underflow part
	Overflow part
_*us*_	Underflow sidestream part
_*is*_	Inlet sidestream part
CP	Centrifugal pump
$V_{x}$	Control valve opening degrees
$P_{x}$	Pressure measurements
$Q_{x}$	Flow rate measurements
PDR	Pressure Difference Ratio
*z*	z-test statistic
$\hat{\mu }$	Individual sample mean
*n*	Number of detected particles
CDF	Cumulative distribution function
KS-test	Kolmogorov-Smirnov test
$d_{KS}$	KS-test statistic
$\hat{CDF}\left(x\right)$	Statistical CDF
$CDF_{BS}$	CDF provided by manufacturer BS-Partikel
*s*	Sample standard deviation
*T*	Chi-square test statistic
$T_{norm}$	Denormalized and decentralized Chi-square test statistic
KL	Kullback-Leibler
$D_{KL}$	KL divergence
P(X)	Data-based probability distribution
Q(X)	Model-based probability distribution
JS	Jensen-Shannon
$D_{JS}$	JS divergence
SSE	Sum of squared errors
$x_{i}$	*i*-th detected particle in the calibration experiment
$C_{i}$	volume concentration at inlet
$C_{u}$	volume concentration at underflow
*e*	Separation efficiency of a segment
